# Investigating the Molecular Epidemiology of Extended-Spectrum β-Lactamase-Producing Enterobacterales (ESBL-E) Among Patients Admitted to the Intensive Care Unit

**DOI:** 10.1093/ofid/ofaf590

**Published:** 2025-10-21

**Authors:** Sima L Sharara, Patricia J Simner, Yehudit Bergman, Emily Jacobs, Suiyini Fiawoo, Eili Y Klein, Sara E Cosgrove, Pranita D Tamma

**Affiliations:** Department of Medicine, Johns Hopkins University School of Medicine, Baltimore, Maryland, USA; Department of Pathology, Johns Hopkins University School of Medicine, Baltimore, Maryland, USA; Department of Pediatrics, Johns Hopkins University School of Medicine, Baltimore, Maryland, USA; Department of Pediatrics, Johns Hopkins University School of Medicine, Baltimore, Maryland, USA; Department of Pediatrics, Johns Hopkins University School of Medicine, Baltimore, Maryland, USA; Department of Medicine, Johns Hopkins University School of Medicine, Baltimore, Maryland, USA; Department of Emergency Medicine, Johns Hopkins University School of Medicine, Baltimore, Maryland, USA; Department of Medicine, Johns Hopkins University School of Medicine, Baltimore, Maryland, USA; Department of Pediatrics, Johns Hopkins University School of Medicine, Baltimore, Maryland, USA

**Keywords:** colonization, CTX-M, ESBL-E, *Escherichia coli*, ICU admission

## Abstract

At a United States hospital, sequencing of ICU rectal surveillance cultures indicated 5% ESBL-E colonization. Of confirmed ESBL isolates, 91% were *Escherichia coli* or *Klebsiella pneumoniae*; 6% carried non-*bla*_CTX-M_ genes. Only 53% of third-generation cephalosporin-resistant Enterobacterales harbored ESBL genes, underscoring the limitations of phenotypic approaches as ESBL surrogates, particularly for non-*E. coli/K. pneumoniae* species.

## Teaser

ICU screening at a U.S. tertiary hospital showed 5% ESBL-Enterobacterales colonization, mainly Escherichia coli and Klebsiella pneumoniae with blaCTX-M. Only half of 3GC-resistant isolates carried ESBL genes, underscoring the limits of phenotypic surrogates in guiding screening and empiric therapy.

Over the past decade, the prevalence of extended-spectrum beta-lactamase-producing Enterobacterales (ESBL-E) has risen markedly in both community and healthcare settings across the United States [1–3]. This increase is driven by a confluence of factors, including horizontal transfer of mobile genetic elements harboring ESBL genes (eg, IncF plasmids) [4], clonal expansion of high-risk bacterial lineages (eg, *Escherichia coli* sequence type 131) [5, 6], consumption of colonized animal products [7], antibiotic overuse [8], lapses in infection prevention measures [9, 10], and human travel and migration from highly endemic regions [11].

The rising incidence of ESBL-E in the United States, including among previously healthy individuals, poses a growing challenge to empiric antibiotic selection for patients admitted to intensive care units (ICUs). Cefepime and piperacillin-tazobactam, widely used as empiric therapies for suspected sepsis, have been associated with inferior clinical outcomes compared with meropenem in patients with invasive ESBL-E infections [12–15].

Understanding local ESBL-E prevalence is essential for guiding empiric antibiotic choices for ICU patients. Because microbiologic species identification is often available before susceptibility results or confirmatory ESBL testing (if performed), knowledge of species-specific ESBL epidemiology can support informed and timely initial treatment decisions. For example, in an ICU with a high prevalence of ESBL-producing *E. coli*, empiric meropenem may be preferred over piperacillin-tazobactam for critically ill patients with suspected gram-negative infections, while awaiting susceptibility results.

While data exist from several European studies [16–18], contemporary estimates of ESBL-E colonization among critically ill patients in the United States remain limited. Furthermore, available prevalence estimates often rely on phenotypic methods—such as growth on selective media or ceftriaxone resistance—which may overestimate the true burden of ESBL-E. While *bla*_CTX-M_ is the predominant ESBL gene [19], the prevalence of other clinically significant genes (eg, *bla*_SHV_) remains underrecognized. Like *bla*_CTX-M,_ non-*bla*_CTX-M_ ESBL-mediated resistance may lead to adverse clinical outcomes with standard empiric antibiotics such as piperacillin-tazobactam [20]. This study aims to characterize the prevalence and molecular epidemiology of ESBL-E colonization among ICU patients at an academic medical center in the United States.

## METHODS

Rectal surveillance swabs were collected for all patients within 7 days of admission to the ICU at The Johns Hopkins Hospital as part of an ongoing infection prevention program to detect ESBL-E across nine ICUs: Pediatric ICU (PICU), Pediatric Cardiac ICU (PCICU), Medical ICU (MICU), Surgical ICU (SICU), Oncology ICU (WICU), Cardiac Care Unit (CCU), Cardiovascular Surgical ICU (CVSICU), Neurocritical ICU (NCCU), and the Comprehensive Transplant Unit (CTU). Samples collected between March and August 2023 were analyzed to characterize the molecular epidemiology of ESBL-E colonization.

Surveillance cultures were collected by trained healthcare personnel using ESwab™ flocked swabs containing 1 mL of liquid Amies medium (Copan Diagnostics, Murrieta, CA). Swabs were plated within 24 hours of collection in the microbiology lab onto HardyCHROM™ ESBL plates (Hardy Diagnostics, Santa Maria, CA) to isolate third-generation cephalosporin-resistant Enterobacterales (3GCRE). 3GCRE isolates recovered on HardyCHROM™ ESBL plates were subsequently plated on CHROMID® CARBA plates (bioMérieux, Inc., Marcy-l'Étoile, France) to assess for carbapenem resistance. Species identification was performed using Matrix-Assisted Laser Desorption Ionization Time-of-Flight mass spectrometry (Bruker, Billerica, MA). Minimum inhibitory concentrations (MICs) of 3GCRE isolates were determined using Sensititre broth microdilution GN7F panels (Thermo Fisher Scientific, Waltham, MA). Isolates were stored at −80°C for further analysis.

Genomic DNA was extracted from the first 3GCRE isolate per species per patient using the PowerSoil Kit (QIAGEN, Valencia, California) for whole genome sequencing (WGS) analysis. For instance, if a patient had *E. coli* isolated at initial ICU admission and the patient was readmitted within 6 months, additional *E. coli* isolates would not be sequenced, but isolates of different species, such as *Klebsiella pneumoniae*, would be sequenced. Extracted DNA was subjected to short-read sequencing using the Illumina MiSeq platform (Illumina, San Diego, California). AREScloud (Ares Genetics, Vienna, Austria) was used for genome assembly, organism identification, sequence type, plasmid type, and antimicrobial resistance gene detection. DIAMOND v1.0.11 was performed on six-frame translated genome assemblies against ARESdb with 60% minimum query coverage and 90% identity for additional antimicrobial resistant gene identification. The NCBI Reference Gene Catalog was used to annotate β-lactamase genes. The study was approved by The Johns Hopkins University School of Medicine Institutional Review Board, with a waiver of informed consent.

## RESULTS

During the 6-month study period, 3,278 index rectal surveillance swabs were collected at ICU admission. Of these, 328 (10%) grew at least one 3GCRE isolate. Among these 328 swabs, 173 (53%) harbored bacteria carrying at least one ESBL gene based on WGS, corresponding to an overall ESBL-E colonization prevalence of 5% across all ICU admissions. The prevalence of ESBL-E colonization varied across ICUs, with the highest percentages observed in the MICU (10%), followed by the CCU (8%), WICU (6%), CTU (6%), PICU (5%), PCICU (4%), SICU (4%), NCCU (4%), and CVSICU (3%). Additional details for the 173 isolates with confirmed ESBL genes regarding ESBL-E species, multilocus sequence type, ESBL genes, *ampC* genes, and carbapenemase genes identified are provided in Supplementary Table 1. Baseline characteristics of participants contributing isolates, stratified by the presence or absence of ESBL-E colonization, are presented in Supplementary Table 2.

**Figure 1.**
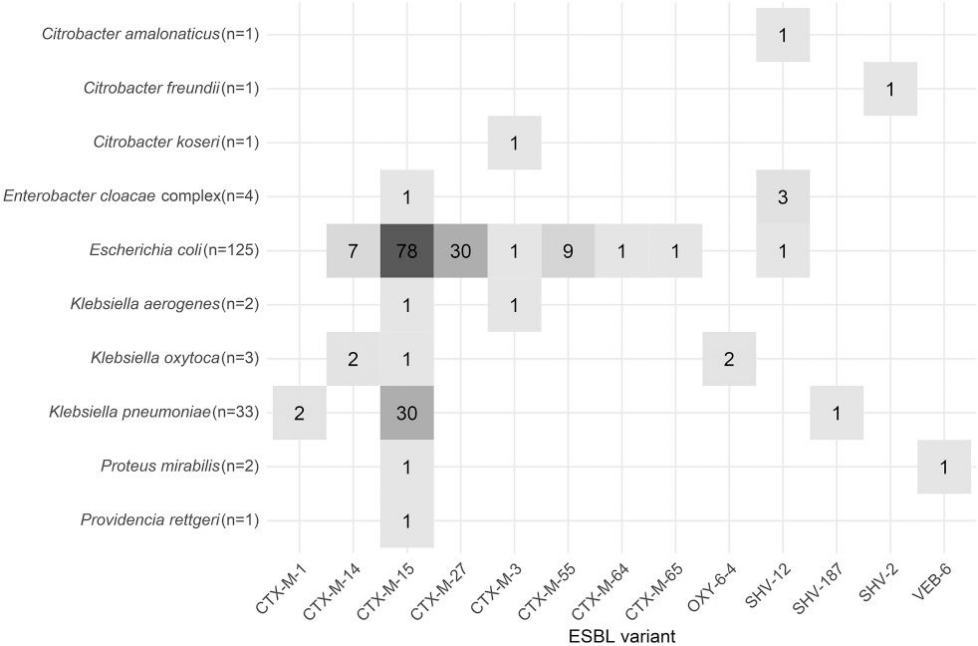
Distribution of ESBL Variants by Bacterial Species.

*E. coli* and *Klebsiella pneumoniae* accounted for most ESBL-producing isolates, comprising 72% (125/173) and 19% (33/173), respectively. Several other bacterial species harbored at least one ESBL gene (Figure 1**)**. Across all species, the distribution of ESBL gene families was as follows: *bla*_CTX-M_ (95%), *bla*_SHV_ (4%), *bla*_OXY_ (1%), and *bla*_VEB_ (1%). No *bla*_PER_, *bla*_TEM_, or *bla*_OXA_ ESBL genes were identified.

Among 125 ESBL-producing *E. coli* isolates, 124 (99%) contained *bla*_CTX-M_ genes, including *bla*_CTX-M-15_ (*n* = 78, 62%), *bla*_CTX-M-27_ (*n* = 30, 24%), *bla*_CTX-M-55_ (*n* = 9, 7%), and *bla*_CTX-M-14_ (*n* = 7, 6%). Three *E. coli* isolates harbored two *bla*_CTX-M_ alleles (ie, two isolates with bla_CTX-M-14_ and bla_CTX-M-15_, one with bla_CTX-M-15_ and bla_CTX-M-27_). Common *E. coli* sequence types were ST131 (*n* = 45, 36%), ST1193 (*n* = 9, 7%), and ST38 (*n* = 7, 6%).

Of the 33 ESBL-producing *K. pneumoniae* isolates, three ESBL alleles were identified: *bla*_CTX-M-15_ (*n* = 30, 91%), *bla*_CTX-M-1_ (*n* = 2, 6%), and *bla*_SHV-187_ (*n* = 1, 3%). Common *K. pneumoniae* sequence types included ST307 (*n* = 5, 15%) and ST45 (*n* = 3, 9%).

Among the 155 3GCRE that were not identified as having an ESBL gene, common species included *Citrobacter freundii* (*n* = 42, 27%), *Enterobacter cloacae* (*n* = 40, 26%), *E. coli* (*n* = 37, 24%), and *Klebsiella aerogenes* (*n* = 14, 9%). Across all 155 isolates without ESBL genes, 85% carried an *ampC* gene. *bla*_CMY_ was the most frequently detected *ampC* gene, followed by *bla*_ACT_ and *bla*_DHA_.

## DISCUSSION

The overall prevalence of ESBL-E colonization amongst ICU admissions at a mid-Atlantic academic medical center was 5%, with the highest rate observed in the MICU (10%). These findings suggest that ESBL-E colonization remains relatively uncommon among ICU patients in the United States—even at a large referral center serving medically complex patients and in a region with substantial international migration. Furthermore, ESBL genes appear largely confined to *E. coli* and *K. pneumoniae*, with 3GCRE of other species rarely harboring ESBL genes, and much more likely to harbor *ampC* genes. Finally, *bla*_CTX-M_ continues to be the dominant ESBL genotype among colonizing isolates.

The Centers for Disease Control and Prevention reported a 48% increase in the prevalence of ESBL-E infections between 2012 and 2017 based on *E. coli* and *K. pneumoniae* isolates exhibiting 3GCRE [1]. Despite these concerning increases, reliable estimates of ESBL-E colonization among patients admitted to United States ICUs remain limited. Data from other regions provide insights into the global burden of ESBL-E colonization. Since 2015, studies in Europe have reported ICU admission colonization rates ranging from 9 to 13% [16–18], with even higher rates observed in Latin America (10–20%) [21], Asia (36–55%) [22, 23], and Africa (55%) [24]. Our findings corroborate that *bla*_CTX-M_ (specifically *bla*_CTX-M-15_) remains the predominant ESBL in the United States [19], with other ESBL genes (eg, *bla*_SHV_, *bla*_TEM,_ bla_OXA_ variants) remaining rare, consistent with other regions of the world [16, 25].

In most United States hospitals, ESBL detection is not routinely performed on clinical or surveillance specimens. Instead, microbiology laboratories often use 3GCRE—particularly in *E. coli* and *K. pneumoniae*—as a surrogate marker for ESBL production. In our study, the use of chromogenic selective media to identify 3GCRE suggested a colonization prevalence of 10%. However, WGS revealed that only 53% of these isolates carried ESBL genes, highlighting the limitations of relying solely on phenotypic resistance as a surrogate for ESBL-E colonization across all Enterobacterales. When confined to only 3GCRE isolates *E. coli* and *K. pneumoniae* isolates, 82% and 85% contained ESBL genes, respectively.

Given the unexpectedly low prevalence of ESBL-E colonization, a risk-based screening approach may offer a more resource-efficient alternative to universal screening. To support this strategy, we are constructing a classification and regression tree to identify patients at high risk of ESBL-E colonization at ICU admission. This model could help focus surveillance efforts on those most likely to benefit, optimizing resource use while maintaining effective detection and infection control.

Our findings are representative of a limited region in the United States, and this work needs to be validated in a larger, geographically diverse cohort. Some patients were inevitably missed during screening. However, baseline demographics, comorbidities, and illness severity were generally comparable between screened and unscreened patients (data not shown), suggesting that missed screens occurred at random.

With ongoing globalization, ESBL-E rates in the United States are expected to rise over time, underscoring the need for regular monitoring of their prevalence and ongoing molecular surveillance to detect early changes in colonization dynamics. Our results provide reassuring insights into current ESBL-E prevalence amongst ICU patients at a large, mid-Atlantic academic medical center in the United States and suggest that (1) ESBL-E colonization at ICU admission is relatively low, (2) phenotypic 3GCR overestimates ESBL-E prevalence if used for Enterobacterales broadly and not limited to *E. coli* and *K. pneumoniae* isolates, and (3) empiric cefepime or piperacillin-tazobactam may remain appropriate for most ICU patients in the absence of specific risk factors for ESBL-E.

## Supplementary Material

ofaf590_Supplementary_Data

## Supplementary Data

Supplementary materials are available at *Open Forum Infectious Diseases* online. Consisting of data provided by the authors to benefit the reader, the posted materials are not copyedited and are the sole responsibility of the authors, so questions or comments should be addressed to the corresponding author.
