# Supplementary material for: Investigating the Molecular Epidemiology of Extended-Spectrum β-Lactamase-Producing Enterobacterales (ESBL-E) Among Patients Admitted to the Intensive Care Unit
Source: Open Forum Infect Dis. 2025 Oct 21;12(10):ofaf590. doi: 10.1093/ofid/ofaf590 (PMC12538287; doi:10.1093/ofid/ofaf590)
Supplement: ofaf590_Supplementary_Data [file ofaf590_supplementary_data.docx]

**Supplemental Table 1.** Molecular Characterization of ESBL-Producing Enterobacterales from ICU Admission Surveillance Swabs at an Academic Medical Center in the United States between March–August 2023

| Isolate | Organism | Multilocus Sequence type | ESBL genes | *ampC* genes^1^ | Carbapenemase genes |
| --- | --- | --- | --- | --- | --- |
| 1 | *Citrobacter amalonaticus* | -- | *bla*_SHV-12_ | -- | - |
| 2 | *Citrobacter freundii* | ST702 | *bla*_SHV-2_ | *bla*_CMY-67_, *bla*_FOX-5_ | *bla*_KPC-2_ |
| 3 | *Citrobacter koseri* | -- | *bla*_CTX-M-3_ | -- | -- |
| 4 | *Enterobacter cloacae* complex | ST148 | *bla*_CTX-M-15_ | *bla*_ACT-74_ | -- |
| 5 | *Enterobacter cloacae* complex | ST92 | *bla*_SHV-12_ | *bla*_ACT-16_ | -- |
| 6 | *Enterobacter cloacae* complex | ST92 | *bla*_SHV-12_ | *bla*_ACT-16_ | -- |
| 7 | *Enterobacter cloacae* complex | -- | *bla*_SHV-12_ | *bla*_ACT-17_ | -- |
| 8 | *Escherichia coli* | ST2003 | *bla*_CTX-M-14_ | *ampC* | -- |
| 9 | *Escherichia coli* | ST38 | *bla*_CTX-M-14_ | *bla*_DHA-1_, *ampC* | -- |
| 10 | *Escherichia coli* | ST2003 | *bla*_CTX-M-14_ | *ampC* | -- |
| 11 | *Escherichia coli* | ST131 | *bla*_CTX-M-14_ | *ampC* | -- |
| 12 | *Escherichia coli* | ST38 | *bla*_CTX-M-14_ | *ampC* | -- |
| 13 | *Escherichia coli* | ST38 | *bla*_CTX-M-14_, *bla*_CTX-M-15_ | *ampC* | *bla*_OXA-244_ |
| 14 | *Escherichia coli* | ST648 | *bla*_CTX-M-14_, *bla*_CTX-M-15_ | *ampC* | -- |
| 15 | *Escherichia coli* | ST13823 | *bla*_CTX-M-15_ | *ampC* | -- |
| 16 | *Escherichia coli* | ST1057 | *bla*_CTX-M-15_ | *ampC* | -- |
| 17 | *Escherichia coli* | ST10 | *bla*_CTX-M-15_ | *ampC* | -- |
| 18 | *Escherichia coli* | ST131 | *bla*_CTX-M-15_ | *ampC* | -- |
| 19 | *Escherichia coli* | ST131 | *bla*_CTX-M-15_ | *ampC* | -- |
| 20 | *Escherichia coli* | ST2325 | *bla*_CTX-M-15_ | *ampC* | -- |
| 21 | *Escherichia coli* | ST131 | *bla*_CTX-M-15_ | *ampC* | -- |
| 22 | *Escherichia coli* | ST131 | *bla*_CTX-M-15_ | *ampC* | -- |
| 23 | *Escherichia coli* | ST131 | *bla*_CTX-M-15_ | *ampC* | -- |
| 24 | *Escherichia coli* | ST131 | *bla*_CTX-M-15_ | *ampC* | -- |
| 25 | *Escherichia coli* | ST973 | *bla*_CTX-M-15_ | *ampC* | -- |
| 26 | *Escherichia coli* | ST131 | *bla*_CTX-M-15_ | *ampC* | -- |
| 27 | *Escherichia coli* | ST1193 | *bla*_CTX-M-15_ | *ampC* | -- |
| 28 | *Escherichia coli* | ST1193 | *bla*_CTX-M-15_ | *ampC* | -- |
| 29 | *Escherichia coli* | ST1193 | *bla*_CTX-M-15_ | *ampC* | -- |
| 30 | *Escherichia coli* | ST131 | *bla*_CTX-M-15_ | *ampC* | -- |
| 31 | *Escherichia coli* | ST95 | *bla*_CTX-M-15_ | *ampC* | -- |
| 32 | *Escherichia coli* | ST44 | *bla*_CTX-M-15_ | *ampC* | -- |
| 33 | *Escherichia coli* | ST648 | *bla*_CTX-M-15_ | *ampC* | -- |
| 34 | *Escherichia coli* | ST405 | *bla*_CTX-M-15_ | *ampC* | -- |
| 35 | *Escherichia coli* | ST410 | *bla*_CTX-M-15_ | *ampC* | -- |
| 36 | *Escherichia coli* | ST1723 | *bla*_CTX-M-15_ | *ampC* | -- |
| 37 | *Escherichia coli* | ST1057 | *bla*_CTX-M-15_ | *ampC* | -- |
| 38 | *Escherichia coli* | ST2279 | *bla*_CTX-M-15_ | *ampC* | -- |
| 39 | *Escherichia coli* | ST58 | *bla*_CTX-M-15_ | *ampC* | -- |
| 40 | *Escherichia coli* | ST773 | *bla*_CTX-M-15_ | *ampC* | -- |
| 41 | *Escherichia coli* | ST69 | *bla*_CTX-M-15_ | *ampC* | -- |
| 42 | *Escherichia coli* | ST131 | *bla*_CTX-M-15_ | *ampC* | -- |
| 43 | *Escherichia coli* | ST131 | *bla*_CTX-M-15_ | *ampC* | -- |
| 44 | *Escherichia coli* | ST131 | *bla*_CTX-M-15_ | *ampC* | -- |
| 45 | *Escherichia coli* | ST5614 | *bla*_CTX-M-15_ | *ampC* | -- |
| 46 | *Escherichia coli* | ST62 | *bla*_CTX-M-15_ | *ampC* | -- |
| 47 | *Escherichia coli* | -- | *bla*_CTX-M-15_ | *ampC* | -- |
| 48 | *Escherichia coli* | ST394 | *bla*_CTX-M-15_ | *ampC* | -- |
| 49 | *Escherichia coli* | ST648 | *bla*_CTX-M-15_ | *ampC* | -- |
| 50 | *Escherichia coli* | ST636 | *bla*_CTX-M-15_ | *ampC* | -- |
| 51 | *Escherichia coli* | ST410 | *bla*_CTX-M-15_ | *bla*_CMY-2_, *ampC* | *bla*_OXA-181_ |
| 52 | *Escherichia coli* | ST1193 | *bla*_CTX-M-15_ | *ampC* | -- |
| 53 | *Escherichia coli* | ST58 | *bla*_CTX-M-15_ | *ampC* | -- |
| 54 | *Escherichia coli* | ST131 | *bla*_CTX-M-15_ | *ampC* | -- |
| 55 | *Escherichia coli* | ST131 | *bla*_CTX-M-15_ | *ampC* | -- |
| 56 | *Escherichia coli* | ST636 | *bla*_CTX-M-15_ | *ampC* | -- |
| 57 | *Escherichia coli* | ST278 | *bla*_CTX-M-15_ | *ampC* | -- |
| 58 | *Escherichia coli* | ST131 | *bla*_CTX-M-15_ | *ampC* | -- |
| 59 | *Escherichia coli* | ST38 | *bla*_CTX-M-15_ | *ampC* | -- |
| 60 | *Escherichia coli* | ST131 | *bla*_CTX-M-15_ | *ampC* | -- |
| 61 | *Escherichia coli* | ST3580 | *bla*_CTX-M-15_ | *ampC* | -- |
| 62 | *Escherichia coli* | ST131 | *bla*_CTX-M-15_ | *ampC* | -- |
| 63 | *Escherichia coli* | ST131 | *bla*_CTX-M-15_ | *ampC* | -- |
| 64 | *Escherichia coli* | ST131 | *bla*_CTX-M-15_ | *ampC* | -- |
| 65 | *Escherichia coli* | ST10 | *bla*_CTX-M-15_ | *ampC* | -- |
| 66 | *Escherichia coli* | ST336 | *bla*_CTX-M-15_ | *ampC* | -- |
| 67 | *Escherichia coli* | ST648 | *bla*_CTX-M-15_ | *ampC* | -- |
| 68 | *Escherichia coli* | ST44 | *bla*_CTX-M-15_ | *ampC* | -- |
| 69 | *Escherichia coli* | ST410 | *bla*_CTX-M-15_ | *ampC* | -- |
| 70 | *Escherichia coli* | ST38 | *bla*_CTX-M-15_ | *ampC* | -- |
| 71 | *Escherichia coli* | ST131 | *bla*_CTX-M-15_ | *ampC* | -- |
| 72 | *Escherichia coli* | ST648 | *bla*_CTX-M-15_ | *ampC* | -- |
| 73 | *Escherichia coli* | ST10 | *bla*_CTX-M-15_ | *ampC* | -- |
| 74 | *Escherichia coli* | -- | *bla*_CTX-M-15_ | *ampC* | -- |
| 75 | *Escherichia coli* | ST8881 | *bla*_CTX-M-15_ | *ampC* | -- |
| 76 | *Escherichia coli* | ST1193 | *bla*_CTX-M-15_ | *ampC* | -- |
| 77 | *Escherichia coli* | ST131 | *bla*_CTX-M-15_ | *ampC* | -- |
| 78 | *Escherichia coli* | ST131 | *bla*_CTX-M-15_ | *ampC* | -- |
| 79 | *Escherichia coli* | ST131 | *bla*_CTX-M-15_ | *ampC* | -- |
| 80 | *Escherichia coli* | ST636 | *bla*_CTX-M-15_ | *ampC* | -- |
| 81 | *Escherichia coli* | ST131 | *bla*_CTX-M-15_ | *ampC* | -- |
| 82 | *Escherichia coli* | ST4121 | *bla*_CTX-M-15_ | *ampC* | -- |
| 83 | *Escherichia coli* | ST131 | *bla*_CTX-M-15_ | *ampC* | -- |
| 84 | *Escherichia coli* | ST131 | *bla*_CTX-M-15_ | *ampC* | -- |
| 85 | *Escherichia coli* | ST155 | *bla*_CTX-M-15_ | *ampC* | -- |
| 86 | *Escherichia coli* | ST131 | *bla*_CTX-M-15_ | *bla*_DHA-1_, *ampC* | -- |
| 87 | *Escherichia coli* | ST95 | *bla*_CTX-M-15_ | *ampC* | -- |
| 88 | *Escherichia coli* | ST4988 | *bla*_CTX-M-15_ | *ampC* | -- |
| 89 | *Escherichia coli* | ST617 | *bla*_CTX-M-15_ | *ampC* | -- |
| 90 | *Escherichia coli* | ST1722 | *bla*_CTX-M-15_, *bla*_CTX-M-27_ | *ampC* | -- |
| 91 | *Escherichia coli* | ST131 | *bla*_CTX-M-27_ | *ampC* | -- |
| 92 | *Escherichia coli* | ST131 | *bla*_CTX-M-27_ | *ampC* | -- |
| 93 | *Escherichia coli* | ST38 | *bla*_CTX-M-27_ | *ampC* | -- |
| 94 | *Escherichia coli* | ST131 | *bla*_CTX-M-27_ | *ampC* | -- |
| 95 | *Escherichia coli* | ST131 | *bla*_CTX-M-27_ | *ampC* | -- |
| 96 | *Escherichia coli* | ST131 | *bla*_CTX-M-27_ | *ampC* | -- |
| 97 | *Escherichia coli* | ST1193 | *bla*_CTX-M-27_ | *ampC* | -- |
| 98 | *Escherichia coli* | -- | *bla*_CTX-M-27_ | *ampC* | -- |
| 99 | *Escherichia coli* | ST131 | *bla*_CTX-M-27_ | *ampC* | -- |
| 100 | *Escherichia coli* | ST131 | *bla*_CTX-M-27_ | *ampC* | -- |
| 101 | *Escherichia coli* | ST58 | *bla*_CTX-M-27_ | *ampC* | -- |
| 102 | *Escherichia coli* | ST131 | *bla*_CTX-M-27_ | *ampC* | -- |
| 103 | *Escherichia coli* | ST131 | *bla*_CTX-M-27_ | *ampC* | -- |
| 104 | *Escherichia coli* | ST131 | *bla*_CTX-M-27_ | *ampC* | -- |
| 105 | *Escherichia coli* | ST131 | *bla*_CTX-M-27_ | *ampC* | -- |
| 106 | *Escherichia coli* | ST38 | *bla*_CTX-M-27_ | *bla*_DHA-1_, *ampC* | -- |
| 107 | *Escherichia coli* | ST1193 | *bla*_CTX-M-27_ | *bla*_CMY-23_, *ampC* | -- |
| 108 | *Escherichia coli* | ST131 | *bla*_CTX-M-27_ | *ampC* | -- |
| 109 | *Escherichia coli* | ST131 | *bla*_CTX-M-27_ | *ampC* | -- |
| 110 | *Escherichia coli* | ST7514 | *bla*_CTX-M-27_ | *ampC* | -- |
| 111 | *Escherichia coli* | ST131 | *bla*_CTX-M-27_ | *ampC* | -- |
| 112 | *Escherichia coli* | ST131 | *bla*_CTX-M-27_ | *ampC* | -- |
| 113 | *Escherichia coli* | ST131 | *bla*_CTX-M-27_ | *ampC* | -- |
| 114 | *Escherichia coli* | -- | *bla*_CTX-M-27_ | *ampC* | -- |
| 115 | *Escherichia coli* | ST10 | *bla*_CTX-M-27_ | *bla*_DHA-1_, *ampC* | -- |
| 116 | *Escherichia coli* | ST131 | *bla*_CTX-M-27_ | *ampC* | -- |
| 117 | *Escherichia coli* | ST998 | *bla*_CTX-M-27_ | *ampC* | -- |
| 118 | *Escherichia coli* | ST131 | *bla*_CTX-M-27_ | *ampC* | -- |
| 119 | *Escherichia coli* | ST1722 | *bla*_CTX-M-27_ | *ampC* | -- |
| 120 | *Escherichia coli* | ST1193 | *bla*_CTX-M-3_ | *ampC* | -- |
| 121 | *Escherichia coli* | ST7804 | *bla*_CTX-M-55_ | *ampC* | -- |
| 122 | *Escherichia coli* | ST8412 | *bla*_CTX-M-55_ | *ampC* | -- |
| 123 | *Escherichia coli* | ST75 | *bla*_CTX-M-55_ | *ampC* | -- |
| 124 | *Escherichia coli* | ST969 | *bla*_CTX-M-55_ | *ampC* | -- |
| 125 | *Escherichia coli* | ST58 | *bla*_CTX-M-55_ | *ampC* | -- |
| 126 | *Escherichia coli* | ST69 | *bla*_CTX-M-55_ | *ampC* | -- |
| 127 | *Escherichia coli* | ST155 | *bla*_CTX-M-55_ | *ampC* | -- |
| 128 | *Escherichia coli* | -- | *bla*_CTX-M-55_ | *ampC* | -- |
| 129 | *Escherichia coli* | ST744 | *bla*_CTX-M-55_ | *ampC* | -- |
| 130 | *Escherichia coli* | ST1193 | *bla*_CTX-M-64_ | *ampC* | -- |
| 131 | *Escherichia coli* | ST2380 | *bla*_CTX-M-65_ | *ampC* | -- |
| 132 | *Escherichia coli* | ST10 | *bla*_SHV-12_ | *ampC* | -- |
| 133 | *Klebsiella aerogenes* | -- | *bla*_CTX-M-15_ | *bla*_DHA-1_ | -- |
| 134 | *Klebsiella aerogenes* | ST190 | *bla*_CTX-M-3_ | *ampC* | -- |
| 135 | *Klebsiella oxytoca* | ST463 | *bla*_CTX-M-14_, *bla*_OXY-6-4_ | -- | -- |
| 136 | *Klebsiella oxytoca* | ST463 | *bla*_CTX-M-14_, *bla*_OXY-6-4_ | -- | -- |
| 137 | *Klebsiella oxytoca* | -- | *bla*_CTX-M-15_ | -- | -- |
| 138 | *Klebsiella pneumoniae* | ST834 | *bla*_CTX-M-1_ | -- | -- |
| 139 | *Klebsiella pneumoniae* | ST834 | *bla*_CTX-M-1_ | -- | -- |
| 140 | *Klebsiella pneumoniae* | ST4081 | *bla*_CTX-M-15_ | -- | -- |
| 141 | *Klebsiella pneumoniae* | ST893 | *bla*_CTX-M-15_ | -- | -- |
| 142 | *Klebsiella pneumoniae* | -- | *bla*_CTX-M-15_ | -- | -- |
| 143 | *Klebsiella pneumoniae* | -- | *bla*_CTX-M-15_ | -- | -- |
| 144 | *Klebsiella pneumoniae* | ST882 | *bla*_CTX-M-15_ | -- | -- |
| 145 | *Klebsiella pneumoniae* | ST101 | *bla*_CTX-M-15_ | -- | -- |
| 146 | *Klebsiella pneumoniae* | ST15 | *bla*_CTX-M-15_ | -- | -- |
| 147 | *Klebsiella pneumoniae* | ST307 | *bla*_CTX-M-15_ | -- | -- |
| 148 | *Klebsiella pneumoniae* | ST307 | *bla*_CTX-M-15_ | -- | -- |
| 149 | *Klebsiella pneumoniae* | -- | *bla*_CTX-M-15_ | -- | -- |
| 150 | *Klebsiella pneumoniae* | ST39 | *bla*_CTX-M-15_ | -- | -- |
| 151 | *Klebsiella pneumoniae* | ST307 | *bla*_CTX-M-15_ | -- | -- |
| 152 | *Klebsiella pneumoniae* | ST1017 | *bla*_CTX-M-15_ | -- | -- |
| 153 | *Klebsiella pneumoniae* | ST1774 | *bla*_CTX-M-15_ | -- | -- |
| 154 | *Klebsiella pneumoniae* | ST45 | *bla*_CTX-M-15_ | -- | -- |
| 155 | *Klebsiella pneumoniae* | ST348 | *bla*_CTX-M-15_ | -- | -- |
| 156 | *Klebsiella pneumoniae* | ST17 | *bla*_CTX-M-15_ | -- | -- |
| 157 | *Klebsiella pneumoniae* | ST268 | *bla*_CTX-M-15_ | -- | -- |
| 158 | *Klebsiella pneumoniae* | ST307 | *bla*_CTX-M-15_ | -- | -- |
| 159 | *Klebsiella pneumoniae* | ST45 | *bla*_CTX-M-15_ | -- | -- |
| 160 | *Klebsiella pneumoniae* | ST323 | *bla*_CTX-M-15_ | -- | -- |
| 161 | *Klebsiella pneumoniae* | ST1128 | *bla*_CTX-M-15_ | -- | -- |
| 162 | *Klebsiella pneumoniae* | ST1774 | *bla*_CTX-M-15_ | -- | -- |
| 163 | *Klebsiella pneumoniae* | ST353 | *bla*_CTX-M-15_ | -- | -- |
| 164 | *Klebsiella pneumoniae* | ST22 | *bla*_CTX-M-15_ | -- | *bla*_OXA-48_ |
| 165 | *Klebsiella pneumoniae* | ST323 | *bla*_CTX-M-15_ | -- | -- |
| 166 | *Klebsiella pneumoniae* | ST45 | *bla*_CTX-M-15_ | -- | -- |
| 167 | *Klebsiella pneumoniae* | ST307 | *bla*_CTX-M-15_ | -- | -- |
| 168 | *Klebsiella pneumoniae* | ST469 | *bla*_CTX-M-15_ | -- | -- |
| 169 | *Klebsiella pneumoniae* | ST17 | *bla*_CTX-M-15_ | -- | *bla*_KPC-2_ |
| 170 | *Klebsiella pneumoniae* | ST29 | *bla*_SHV-187_ | *bla*_CMY-2_ | -- |
| 171 | *Proteus mirabilis* | -- | *bla*_CTX-M-15_ | -- | -- |
| 172 | *Proteus mirabilis* | -- | *bla*_VEB-6_ | -- | -- |
| 173 | *Providencia rettgeri* | -- | *bla*_CTX-M-15_ | -- | -- |

--: Not detected; ^1^The chromosomal *ampC* gene in *E. coli* is also referred to as the *bla*_EC_ gene.

| **Supplemental Table 2.** Patient Characteristics at ICU Admission Stratified by Third-Generation Cephalosporin-Resistant Enterobacterales (3GCRE) Surveillance Status | | |
| --- | --- | --- |
| **Patient characteristics** | **Positive 3CGRE Surveillance Swab**  **(n=328)** | **Negative 3CGRE Surveillance Swab**  **(n=2951)** |
| Age, mean (SD) | 48.9 (25.6) | 49.6 (25.1) |
| Female | 162 (49.4%) | 1335 (45.2%) |
| Race/Ethnicity |  |  |
| White | 149 (45.4%) | 1620 (54.9%) |
| Black | 105 (32.0%) | 891 (30.2%) |
| Hispanic | 20 (6.1%) | 166 (5.6%) |
| Other | 54 (16.5%) | 274 (9.3%) |
| Vasopressor requirement | 97 (29.6%) | 677 (22.9%) |
| Mechanical ventilation | 96 (29.3%) | 854 (28.9%) |
| Charlson Comorbidity Score, mean (SD) | 4.4 (3.2) | 3.5 (3.0) |
| Cerebrovascular disease | 60 (18.3%) | 576 (19.5%) |
| Congestive heart failure | 81 (24.7%) | 646 (21.9%) |
| Chronic pulmonary disease | 90 (27.4%) | 604 (20.5%) |
| Dementia | 40 (12.2%) | 241 (8.2%) |
| Diabetes without complications | 89 (27.1%) | 643 (21.8%) |
| Diabetes with complications | 65 (19.8%) | 438 (14.8%) |
| HIV infection | 3 (0.9%) | 35 (1.2%) |
| Hemiplegia or paraplegia | 38 (11.6%) | 212 (7.2%) |
| Malignancy | 74 (22.6%) | 656 (22.2%) |
| Mild liver disease | 59 (18.0%) | 356 (12.1%) |
| Moderate or severe liver disease | 28 (8.5%) | 113 (3.8%) |
| Renal disease (mild or moderate) | 83 (25.3%) | 567 (19.2%) |
| Renal disease (severe) | 50 (15.2%) | 276 (9.4%) |
| Peptic ulcer disease | 11 (3.4%) | 42 (1.4%) |
| Peripheral vascular disease | 82 (25.0%) | 580 (19.7%) |
| Admitted from a health care facility | 121 (36.9%) | 882 (29.9%) |

Continuous variables are presented as mean (standard deviation); categorical variables are presented as N (%).
